# A Self-Supervised Pre-Trained Transformer Model for Accurate Genomic Prediction of Swine Phenotypes

**DOI:** 10.3390/ani15172485

**Published:** 2025-08-24

**Authors:** Weixi Xiang, Zhaoxin Li, Qixin Sun, Xiujuan Chai, Tan Sun

**Affiliations:** 1 Agricultural Information Institute, Chinese Academy of Agricultural Sciences, Beijing 100081, China; 82101222481@caas.cn (W.X.); lizhaoxin@caas.cn (Z.L.); sunqixin@caas.cn (Q.S.); suntan@caas.cn (T.S.); 2 Key Laboratory of Agricultural Big Data, Ministry of Agriculture and Rural Affairs, Beijing 100081, China

**Keywords:** genomic prediction, phenotype prediction, transformer, self-supervised learning, non-additive effects, swine

## Abstract

Accurate genomic prediction of complex phenotypes is crucial for accelerating genetic progress in swine breeding. However, conventional methods like Genomic Best Linear Unbiased Prediction (GBLUP) face limitations in capturing complex non-additive effects that contribute significantly to phenotypic variation, restricting the potential accuracy of phenotype prediction. To address this challenge, we introduce a novel framework based on a self-supervised, pre-trained encoder-only Transformer model. Its core novelty lies in tokenizing SNP sequences into non-overlapping 6-mers (sequences of 6 SNPs), enabling the model to directly learn local haplotype patterns instead of treating SNPs as independent markers. The model first undergoes self-supervised pre-training on the unlabeled version of the same SNP dataset used for subsequent fine-tuning, learning intrinsic genomic representations through a masked 6-mer prediction task. Subsequently, the pre-trained model is fine-tuned on labeled data to predict phenotypic values for specific economic traits. Experimental validation demonstrates that our proposed model consistently outperforms baseline methods, including GBLUP and a Transformer of the same architecture trained from scratch (without pre-training), in prediction accuracy across key economic traits. This outperformance suggests the model’s capacity to capture non-linear genetic signals missed by linear models. This research contributes not only a new, more accurate methodology for genomic phenotype prediction but also validates the potential of self-supervised learning to decipher complex genomic patterns for direct application in breeding programs. Ultimately, this approach offers a powerful new tool to enhance the rate of genetic gain in swine production by enabling more precise selection based on predicted phenotypes.

## Simple Summary

Predicting complex genetic traits is essential for improving swine-breeding programs, but traditional methods face limitations. This study introduces a novel deep learning framework, using a Transformer model, to more accurately predict swine phenotypes. The model first learns the fundamental patterns of the pig genome from genetic data and is then fine-tuned to predict key economic traits. Our results show this method outperforms existing approaches, like GBLUP. This enhanced accuracy provides breeders with a powerful tool for selecting superior animals, potentially accelerating genetic gain and delivering substantial economic benefits to the swine industry.

## 1. Introduction

Precision breeding represents a critical driver of economic efficiency and genetic advancement in modern animal husbandry, with its impact being particularly profound within the global swine industry [1]. The relentless pursuit of accelerated genetic improvement is fueled by the need to enhance economically important traits such as growth rate, feed efficiency, and disease resistance to meet growing consumer demand sustainably [2,3]. Genomic selection (GS) has emerged as the cornerstone of this endeavor, revolutionizing the field by leveraging whole-genome marker information to predict phenotypic values with greater accuracy [4]. By enabling earlier and more precise selection decisions, GS has surpassed traditional pedigree-based methods, becoming an indispensable tool for modern breeding programs [5]. However, despite these advances, a significant and persistent challenge remains: conventional genomic prediction methods are approaching a performance plateau, limiting the potential for further accelerating genetic gain and indicating a pressing need for more powerful analytical approaches [6,7].

The primary limitation of existing methodologies lies in their inherent inability to fully decipher the complex, non-additive genetic architecture that governs most economically relevant traits [7]. Conventional methods, such as Genomic Best Linear Unbiased Prediction (GBLUP) and various Bayesian approaches, have proven effective in capturing additive genetic effects [4,8]. Yet, they often operate on simplifying assumptions, such as a common variance for all SNP effects, which hinders their capacity to model the intricate web of epistatic interactions between genes and the long-range dependencies that exist within genomic sequences [4]. Consequently, a substantial portion of the heritable variation remains uncaptured, creating a clear methodological gap [6]. While the field has explored more sophisticated techniques, including machine learning methods like Lasso and random forests, and even early deep learning architectures like Convolutional and Recurrent Neural Networks, these models have also shown limitations [7,9,10]. They may offer improvements in handling high dimensionality, but often fall short of comprehensively modeling the hierarchical and spatially ordered nature of genomic information (i.e., the linear arrangement of markers along a chromosome), thus failing to fully exploit the rich data encoded in an organism’s DNA [11,12].

This research addresses this methodological void by introducing a novel framework built upon the Transformer architecture, a deep learning model that has shown revolutionary success in natural language processing [13]. We posit that the self-attention mechanism at the core of the Transformer is uniquely suited to overcome the limitations of prior models by effectively capturing complex contextual relationships and long-range dependencies within sequential data, making it an ideal candidate for genomic analysis [14,15]. Furthermore, we leverage the power of self-supervised learning (SSL), a paradigm that enables models to learn rich, effective representations from genomic data itself, used in an unlabeled fashion [16,17]. This is particularly advantageous in genomics, where unlabeled SNP sequence data is abundant, while corresponding high-quality phenotype data is often scarce and expensive to acquire [16].

Therefore, we advance the central scientific hypothesis that an encoder-only Transformer model, when first pre-trained on the unlabeled SNP sequences from the datasets used in this study via a self-supervised masked prediction task, can learn the fundamental syntax of the genome, including linkage disequilibrium patterns, local haplotypes, and complex interactive effects [15,18]. We hypothesize that this pre-learned knowledge provides a robust initialization, enabling the model, after fine-tuning on specific labeled datasets, to predict phenotypic values for key economic traits with substantially greater accuracy than both established baseline methods and a Transformer model trained from scratch. The main contributions of this work are thus threefold: the development of an innovative methodology applying a self-supervised, pre-trained Transformer to genomic prediction in pigs; a comprehensive demonstration of its superior predictive performance against a suite of existing models; and an exploration of the model’s interpretability to gain novel insights into the genomic features driving its predictions [19,20]. This approach promises a powerful new tool for genomic selection, potentially unlocking faster and more substantial genetic gains in swine production.

## 2. Materials and Methods

To systematically validate the effectiveness of the self-supervised pre-trained Transformer model proposed in this study in addressing the challenges of complex non-additive effects and long-range dependencies, we have designed the following experimental protocol for evaluation and validation. This section first introduces the key data resources used in the study, including genomic and phenotypic value data from multiple swine populations (Section 2.1). Subsequently, to ensure the transparency and reproducibility of our research, we will detail the specific model architecture (Section 2.2), the two-stage training strategy (Section 2.3), and the complete experimental design, which includes baseline models and evaluation metrics (Section 2.4).

### 2.1. Dataset

#### 2.1.1. Data Source and Description

This study utilized two publicly available swine population datasets. For improved clarity and traceability, the datasets were renamed to reflect their origin and core content. The first dataset is the PIC Genomic Dataset (PIC-GD), which was made public by PIC (a Genus Company, Hampshire, UK ) to serve as a common resource for the scientific community, particularly for comparing and benchmarking genomic prediction methods [21]. The data originates from a single PIC nucleus pig line and includes 3534 animals. Its components include high-density genotype data from the Illumina PorcineSNP60 chip, filtered to 52,842 SNPs, and a complete pedigree file. Crucially, the dataset provides recorded phenotypic values for five distinct purebred traits. According to the data provider, these traits exhibit a wide range of heritabilities (from 0.07 to 0.62), which provides an excellent basis for evaluating the model’s performance on traits with varying degrees of genetic control. All necessary pedigree and fixed effect information for traditional genetic evaluation were also included.

The second dataset is the Huazhong Agricultural University—Piglet Mortality (HZA-PMB) at Birth (HZA-PMB) [22]. This dataset was established to characterize the genetic and genomic fundamentals of sow reproductive traits. The data was collected from multi-breed populations of Yorkshire, Landrace, and Duroc sows. The prediction targets for this dataset are the recorded phenotypic values for Piglet Mortality (HZA-PMB) at birth across the first three parities. Each parity is treated as a distinct, albeit related, trait. These traits exhibit low heritability (estimated between 0.063 and 0.114) [22]. All individuals were genotyped using the Illumina PorcineSNP60 BeadChip.

For both datasets, the provided heritability estimates are critical, as they represent the proportion of phenotypic variance attributable to additive genetic effects and thus serve as a theoretical performance benchmark for linear models like GBLUP (Table 1).

#### 2.1.2. Data Preprocessing

The raw SNP data underwent a standard quality control (QC) procedure. SNPs were removed if their call rate was below 95% (missing rate > 5%), their minor allele frequency (MAF) was less than 1%, or if they significantly deviated from Hardy–Weinberg equilibrium (HWE p < $10^{-6}$). Furthermore, individuals with more than 10% missing genotypes were excluded from the analysis. After QC, the remaining SNPs were numerically encoded, where 0, 1, and 2 represented homozygous for the major allele, heterozygous, and homozygous for the minor allele, respectively. This encoding process is not strand-aware, as the input SNP data was already standardized to reference and alternative alleles. The very few missing genotypes remaining post-QC (<<0.1%) were imputed using the mean genotype of the respective SNP locus.

To prepare the data for the Transformer model, each individual’s complete SNP sequence was processed into a non-overlapping sequence of 6-mer tokens, up to a maximum length of 10,240. A special [CLS] token was prepended to each sequence, intended to be used as an aggregate representation for downstream prediction tasks. Sequences shorter than this defined maximum length were padded with a special [PAD] token to ensure uniform input size [15].

### 2.2. Model Architecture

**Figure 1:**
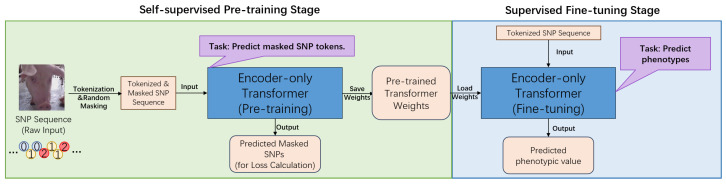
Schematic diagram of the masked SNP prediction (MSP) task for self-supervised pre-training, and supervised fine-tuning (SFT).

**Figure 2:**
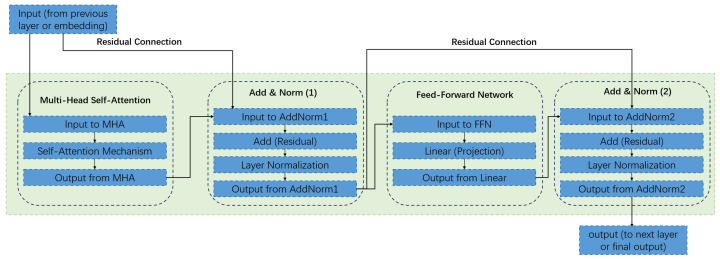
Detailed structure of a single Transformer encoder layer, illustrating the multi-head self-attention (MHSA) and feed-forward network (FFN) sub-layers with residual connections and layer normalization.

The core of this research is a novel computational framework centered on an encoder-only Transformer architecture [13], specifically designed to address the challenges of genomic prediction in swine (Figure 1 and Figure 2). This framework conceptualizes SNP sequences as a form of biological language [15,18], allowing the model to learn the complex mapping from genotype to phenotype. Its architecture is engineered to overcome the limitations of conventional methods in capturing non-additive effects and long-range dependencies within the genome [13,14].

The model begins by transforming raw numerical SNP data into a high-dimensional vector space through a multi-component input representation layer. This involves creating learnable SNP token embeddings for each unique allele value and special tokens, which are then combined with position embeddings that inform the model of the sequential order of SNPs along the chromosome. This rich representation serves as the input for the model’s core engine: a stack of twelve identical Transformer encoder layers. Each layer is composed of two primary sub-modules. The first, a multi-head self-attention mechanism, is crucial for modeling complex genetic architecture. It allows the model to weigh the importance of all other SNPs in a sequence when processing a single SNP, thereby effectively capturing both local and distant interactions, such as epistasis [13]. This is followed by a fully connected feed-forward network, which provides further non-linear transformation to enhance the model’s expressive power.

A key innovation of our framework is its two-stage training paradigm, which utilizes different task-specific output heads. In the initial self-supervised pre-training phase, a simple linear head is added to predict randomly masked SNPs from the genomic portion of the datasets, treated as unlabeled. This task compels the model to learn the intrinsic patterns of the swine genome, such as linkage disequilibrium and allele frequencies, without any phenotypic information [15]. For the subsequent supervised fine-tuning stage, this pre-training head is replaced with a regression head. This head uses the aggregated representation from the special [CLS] token to predict final phenotypic values for specific economic traits using labeled data. This pre-training and fine-tuning approach represents a significant novelty, as it enables the model to develop a deep understanding of genomic structure, leading to demonstrably superior prediction accuracy compared to baseline models like GBLUP or a Transformer trained from scratch [7,23].

Ultimately, the framework is designed to deliver both methodological and applied contributions to the field of computational breeding. Methodologically, it introduces a new, powerful, and scalable deep learning model that leverages large-scale unlabeled data, offering a novel paradigm for genomic prediction that can be adapted to other species. From an applied perspective, the enhanced prediction accuracy directly translates to the potential for higher genetic gain. By enabling more precise selection decisions, this model can help accelerate genetic improvement in swine-breeding programs, leading to significant productivity and economic benefits for the industry. Furthermore, interpretability analyses of the model’s attention patterns may offer new insights into the genetic architecture of complex traits by highlighting novel genomic regions of interest [19,24].

### 2.3. Training Strategy

The model’s training is conducted in a two-stage paradigm: a self-supervised pre-training phase followed by a supervised fine-tuning phase. This approach is designed to first let the model learn robust representations of genomic architecture from large-scale data and then adapt this knowledge to specific prediction tasks [11,16].

**Figure 3:**
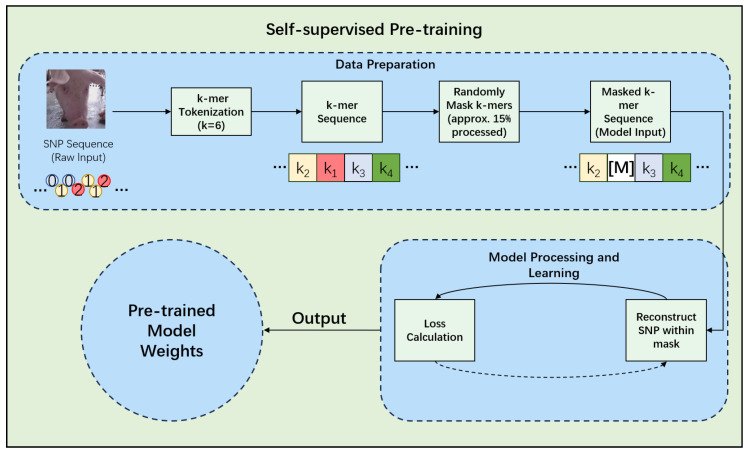
Illustration of the masked 6-mer prediction (M6P) task for self-supervised pre-training. A sample 6-mer SNP sequence is shown with some positions masked, which the model learns to predict.

The self-supervised pre-training phase employs a masked 6-mer prediction (M6P) objective, a critical methodological shift from masking individual SNPs (Figure 3). This strategy is predicated on the biological principle that SNPs are often inherited in blocks (haplotypes). By tokenizing the genome into non-overlapping 6-mer sequences, the model is tasked with learning local haplotype patterns directly, which also significantly reduces the input sequence length for computational efficiency [15,17]. During this stage, 15% of the 6-mer tokens in each sequence are randomly selected for prediction. Of these, 80% are replaced with a special [MASK] token, 10% are replaced with a random 6-mer token from the vocabulary, and the remaining 10% are left unchanged [15]. This pre-training utilizes the complete genotypic datasets from both the PIC Genomic Dataset (PIC-GD) and the Huazhong Agricultural University—Piglet Mortality (HZA-PMB) at Birth (HZA-PMB) populations, without relying on any phenotype labels. This allows the model to learn a generalized understanding of swine genomic structure from diverse genetic backgrounds. The model is trained to predict the original 6-mer tokens at the masked positions using a cross-entropy loss function, with the AdamW optimizer and a learning rate schedule involving a warm-up and subsequent decay [13].

In the supervised fine-tuning phase, the pre-trained model is adapted for the regression task of predicting phenotypic values. The weights of the Transformer encoder, learned during pre-training, are used to initialize the model (Table 2). This transfer learning strategy leverages the learned genomic knowledge for specific downstream tasks, such as predicting the phenotypic values from the PIC-GD dataset or the HZA-PMB dataset [16]. For this stage, only individuals with both genotype data and corresponding phenotypic records are used. The dataset is carefully split into training, validation, and testing sets, using pedigree information from the PIC-GD dataset to prevent data leakage by ensuring closely related individuals are not distributed across different sets [2,25]. The model is then trained to minimize the Mean Squared Error (MSE) between its predictions and the true values. A smaller learning rate is typically used with the AdamW optimizer to preserve the knowledge acquired during pre-training. The validation set plays a crucial role in model selection based on metrics: the coefficient of determination (R^2^) and the Mean Squared Error (MSE), as well as for implementing an early stopping strategy to prevent overfitting [26].

### 2.4. Experimental Design

To systematically evaluate the performance of our proposed pre-trained Transformer model, a comprehensive experimental design was established. The model’s efficacy was benchmarked against several baseline models chosen to represent a spectrum of current techniques in the field. These included Genomic Best Linear Unbiased Prediction (GBLUP), which serves as the industry-standard linear model [4,5]; traditional machine learning algorithms, specifically Lasso and LightGBM (LGBM), to represent non-deep learning approaches capable of handling high-dimensional data [6,9]; and a Transformer model trained entirely from scratch. This “from-scratch” Transformer, possessing an identical architecture to our proposed model but without the self-supervised pre-training phase, was included specifically to quantify the contribution of the pre-training strategy to the final prediction performance [16]. All experiments were conducted on a high-performance computing cluster equipped with two NVIDIA A6000 GPUs (48 GB of memory each). The self-supervised pre-training phase required approximately 72 h, while fine-tuning for each trait was completed in about 4 h, with a peak GPU memory usage of around 50 GB during pre-training.

To assess the model’s ability to predict phenotypic values for future generations, a forward validation strategy based on pedigree information was employed. This approach mimics the real-world application of genomic selection, where data from ancestral populations are used to predict the genetic merit of younger individuals. Specifically, we partitioned the dataset according to parent–offspring relationships, ensuring a temporal split. The oldest 80% of individuals constituted the training set, the subsequent 10% formed the validation set, and the youngest 10% of individuals were held out as the final test set. This across-generation validation scheme prevents any information from descendants being used to train or tune the model, thus providing an unbiased estimate of the model’s forward predictive power. This setup is inherently more challenging than a random split, as it directly evaluates the model’s ability to generalize to new, non-overlapping generations, a scenario where the reliance on close relatives for prediction is minimized.

Optimal configurations for each model were identified through a systematic Grid Search methodology. For each hyperparameter combination, models were trained on the training set, and their performance was assessed on the validation set using the coefficient of determination (R^2^). The parameter set yielding the highest R^2^ on the validation set was selected for the final model configuration. The test set was excluded entirely during all training and tuning phases and was used only once for the final, unbiased performance assessment. To ensure exact reproducibility of our results, a fixed random seed (seed = 42) was used for all stochastic processes, including model weight initialization and the random masking operations during pre-training.

The model’s training objective was to minimize the Mean Squared Error (MSE) loss function between the predicted and observed phenotypic values. The final predictive performance of all models on the test set was quantified using two primary metrics: the coefficient of determination (R^2^), to measure the proportion of variance in the observed phenotypic values that is predictable from the model, and the MSE, to quantify the overall magnitude of prediction error [25]. Finally, to gain insight into the model’s decision-making process, we analyzed the Transformer’s attention weights to identify specific SNP regions the model deemed most important for prediction, aiming to uncover potentially novel, biologically relevant genomic loci [19,20].

## 3. Results

This section systematically presents the performance evaluation of our proposed pre-trained Transformer model for swine phenotypic value prediction, benchmarked against a suite of baseline methods. The evaluation adheres to the core principles of “Experimental Design & Validation” outlined in the guide to computational breeding research, aiming to rigorously demonstrate the model’s efficacy in achieving more accurate predictions on real-world genomic data [3,6].

### 3.1. Pre-Training Performance

**Figure 4:**
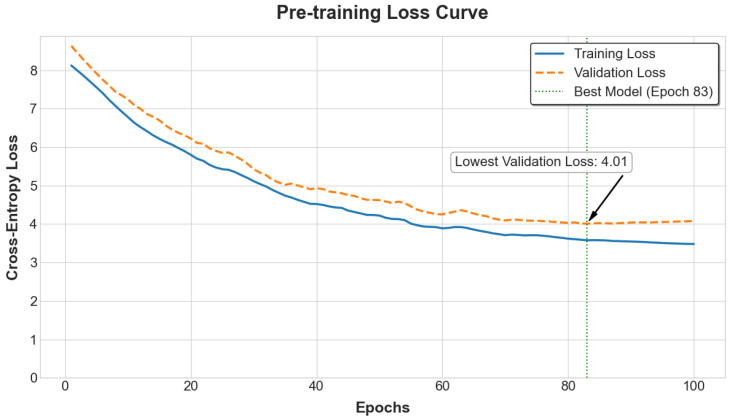
Loss curve for the masked 6-mer SNP prediction task during the self-supervised pre-training phase, showing convergence over training steps.

The model first underwent a self-supervised pre-training phase on the complete, unlabeled genomic data from both the PIC-GD and HZA-PMB datasets. The pre-training objective was Masked 6-mer Prediction [15]. As illustrated in Figure 4, the training and validation losses steadily decreased, reaching convergence on the validation set at 83 epochs with a final cross-entropy loss of 4.01. This indicates that the model successfully learned meaningful, underlying representations of genomic structure from the large-scale SNP sequences, establishing a robust foundation for the subsequent supervised fine-tuning tasks [11,15,16,17].

### 3.2. Phenotypic Prediction Performance

Following pre-training, the model was initialized with the learned weights and then fine-tuned on two distinct labeled datasets to predict phenotypic values for traits with different genetic architectures: (1) Growth Trait (PIC-GD): Five key economic trait with moderate-to-high heritability from the PIC-GD purebred dataset [21]. (2) Piglet Mortality (HZA-PMB): Three complex, low-heritability reproductive trait from the HZA-PMB multi-breed dataset [22].

**Figure 5:**
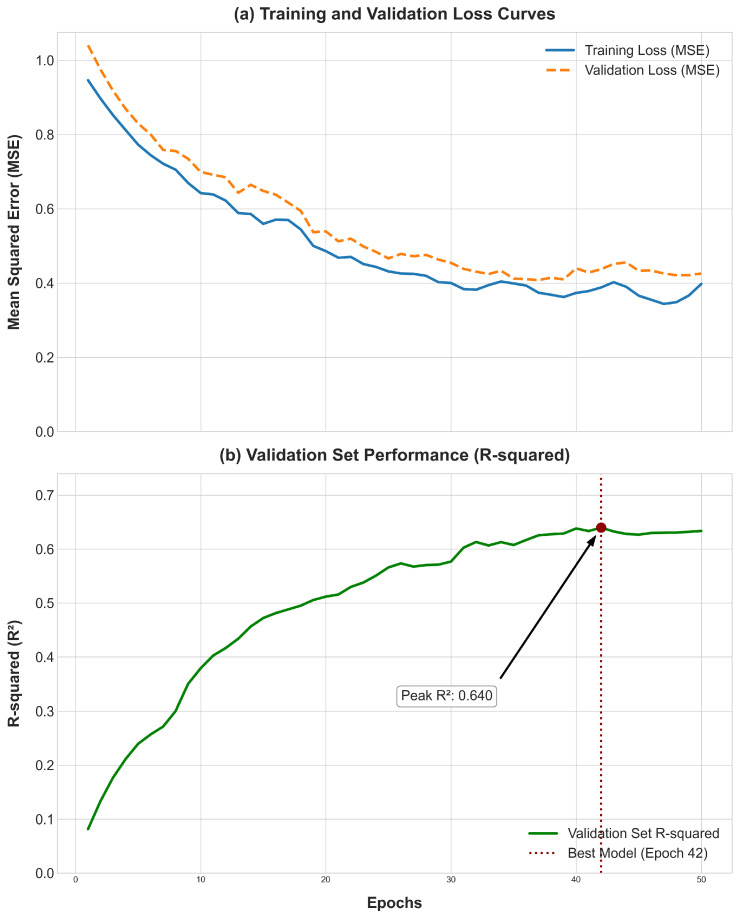
Training dynamics during the fine-tuning phase on PIC-GD T5. (**a**) Training and validation loss (MSE) curves. (**b**) Validation set performance metric (R-squared correlation) over epochs for key traits.

The model’s final predictive performance on the test sets is summarized as follow. For PIC-GD, the model achieved a average R-squared of 0.3281, and for HZA-PMB, the coefficient of determination (R^2^) was 0.0820. The fine-tuning dynamics for Growth Trait (PIC-GD) T5 are detailed in Figure 5, where the training and validation Mean Squared Error (MSE) show stable convergence, and the validation set R-squared peaks at 0.640 around epoch 42, confirming an effective and stable fine-tuning process.

### 3.3. Comparison with Baseline Models

To validate the superiority of our approach, we benchmarked its performance against a suite of baseline models, including the industry-standard GBLUP [4], traditional machine learning methods (Lasso, LightGBM), other deep learning architectures (CNN, LSTM) [6,9], and an identical Transformer model trained from scratch (without pre-training).

As shown in Table 3, the pre-trained Transformer consistently demonstrated the best performance. For average performance on PIC-GD, our model achieved an R-squared of 0.3281, substantially outperforming GBLUP and the from-scratch Transformer.

For the more complex, low-heritability Piglet Mortality (HZA-PMB), our model’s R-squared of 0.0820 was also higher than all baselines, including GBLUP.

**Figure 6:**
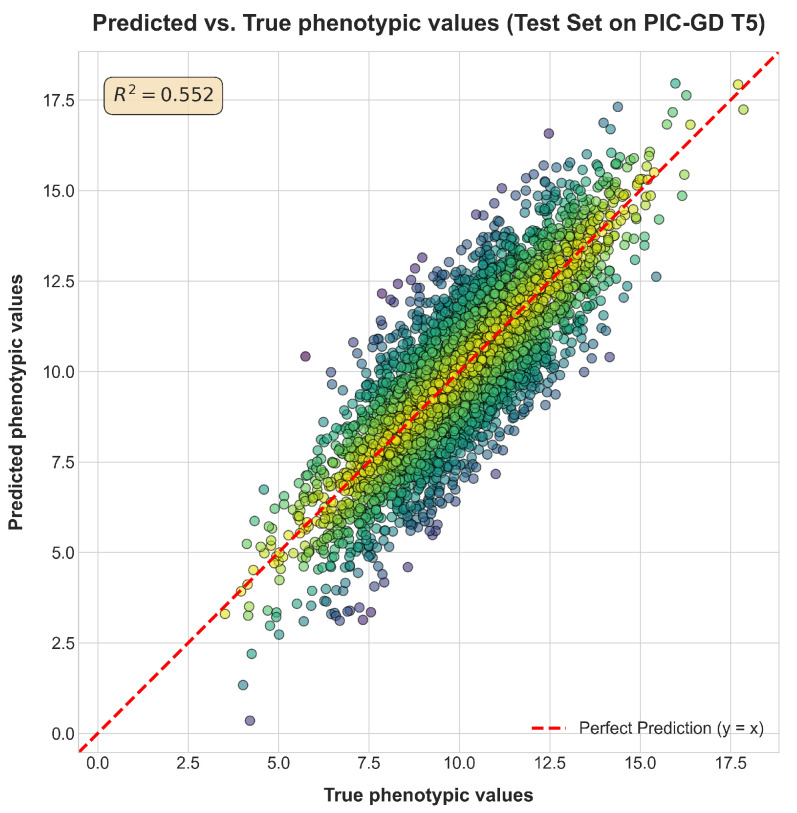
Scatter plot of predicted vs. true phenotypic values on the test set for PIC-GD T5. The y = x line and coefficient of determination are shown. The color of each data point represents the magnitude of the prediction residual (the absolute difference between the predicted and true values), with darker colors indicating a greater deviation from the perfect prediction line (y = x).

These results robustly demonstrate that (1) the Transformer architecture is inherently more capable of capturing complex genetic effects than linear models and other tested architectures [13,14], and (2) self-supervised pre-training is the critical step that unlocks this potential by providing a powerful initialization based on general genomic knowledge [15,16,18]. Figure 6 visually confirms the strong agreement between predicted and true phenotypic values (R^2^ = 0.552) for PIC-GD T5.

### 3.4. Ablation Study

To quantify the contribution of key components, particularly self-supervised pre-training, we conducted ablation studies. As highlighted in the comparison in Section 3.3, removing the pre-training phase resulted in a substantial R-squared decrease on both PIC-GD and HZA-PMB dataset. This finding quantitatively confirms the impact of pre-training on final model performance.

### 3.5. Model Interpretation Insights

To understand how our model achieves superior accuracy, we investigated its internal decision-making process by visualizing the self-attention mechanism [19,20]. This analysis aimed to verify the Transformer’s theoretical ability to overcome the limitations of conventional models by capturing long-range dependencies within the genome [13]. To ground these computational findings in biology, we cross-validated the high-attention regions identified by the model with the Pig Quantitative Trait Loci (QTL) database (Pig QTLdb) [27], a strategy advocated for ensuring biological plausibility.

A core tenet of our research is that the Transformer’s self-attention mechanism can capture long-range dependencies within the genome that are biologically significant [13]. If these dependencies are meaningful, the model should focus on regions that correspond to known functional genomic elements, such as QTLs. Taking Piglet Mortality (HZA-PMB) in the HZA-PMB dataset as an example, our model identified a high-attention region on chromosome 13. This finding aligns remarkably with published research that has previously identified QTLs related to the number of stillborn piglets on chromosomes 5 and 13 [28,29].

**Figure 7:**
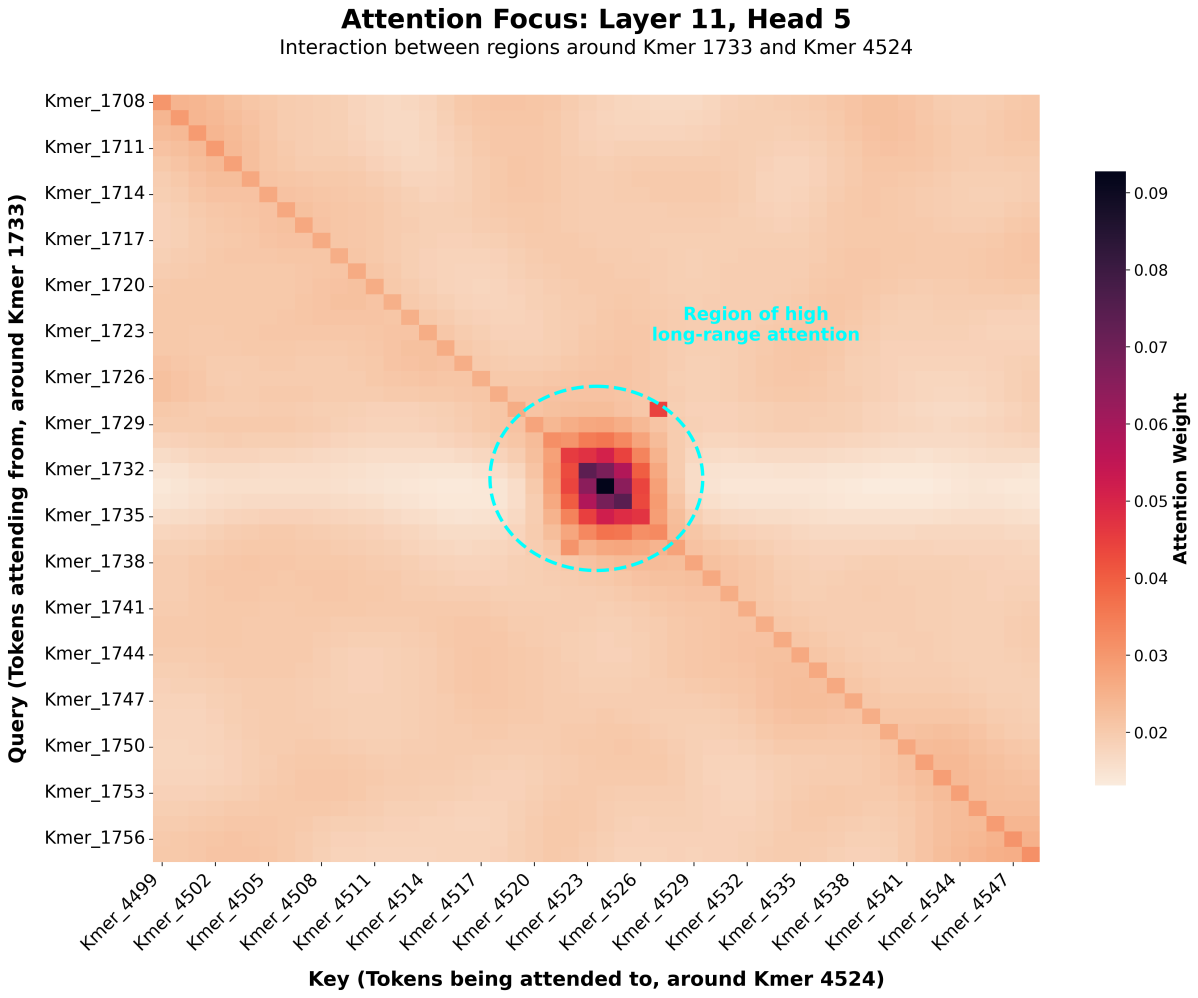
Visualization of attention scores between k-mer tokens for Trait 1 prediction in the PIC-GD dataset. The x- and y-axes represent the sequential index of tokens along the chromosome, not physical base-pair coordinates. This visualization reveals long-range dependencies learned by the model.

As shown in attention maps (an example concept depicted in Figure 7), when predicting a trait, the model simultaneously assigns high attention scores to multiple, physically distant genomic regions. It is important to clarify that our model operates not on individual SNPs but on k-mer tokens, where each token represents a sequence of SNPs. Consequently, the attention mechanism identifies these tokenized regions as important, which precludes the pinpointing of individual SNP loci from this level of analysis. This demonstrates that the model is not just relying on local linkage disequilibrium but is actively identifying and utilizing relationships between distal loci, which is crucial for modeling complex epistatic interactions [7,30]. The alignment of our model’s predictions with established biological markers strongly suggests that it is not merely capturing statistical noise but is learning to identify genomic loci with known relevance to the predicted trait. This capability not only validates the effectiveness of our model but also establishes it as a powerful hypothesis-generation tool for discovering novel candidate gene regions.

This capability to capture dependencies across vast genomic distances is a primary reason for its enhanced predictive power [14]. The successful identification of regions corresponding to known interacting Quantitative Trait Loci (QTLs) [31,32] suggests the model is learning biologically meaningful genetic networks.

## 4. Discussion

This study was designed to address a critical “methodological gap” in genomic prediction: the inherent limitation of conventional methods like GBLUP in capturing the complex non-additive effects and long-range dependencies that govern quantitative traits [4,7]. To overcome this, we proposed and validated a novel framework centered on a self-supervised, pre-trained Transformer model [13,15,16]. This discussion will first interpret our findings by directly addressing the core research hypothesis, then summarize the study’s primary methodological and applied contributions, explore the model’s potential for generating biological insights, and finally, acknowledge its limitations and outline promising avenues for future research.

### 4.1. Performance and Comparison with Baselines

Our central scientific hypothesis was that a Transformer model, pre-trained on genomic data in a self-supervised manner, could learn the complex, non-linear mapping from genotype to phenotype [15,18]. A key aspect of our study design was the use of recorded phenotypes as the prediction target. This strategy directly addresses the ultimate goal of a breeding program: to select animals that will exhibit superior performance. We posited that by capturing non-linear signals present in the raw phenotype—signals arising from non-additive genetic effects that are inherently missed by the linear models like GBLUP—our approach would yield superior predictive accuracy. Crucially, we tested this hypothesis under a realistic forward validation scenario, designed to assess the model’s ability to predict the phenotypic outcomes of future generations from ancestral data [9,12].

The experimental results provide strong evidence for this hypothesis. As shown in Table 3, our pre-trained Transformer model consistently achieved the highest prediction accuracy (measured by R^2^) on the held-out test set of the youngest individuals. A key observation is the performance of the industry-standard GBLUP model [4,33]. For traits with known heritability (*h*^2^), GBLUP’s predictive accuracy (R^2^) is theoretically capped by *h*^2^, as it primarily models the additive genetic component of phenotypic variance [4]. Our results align with this expectation; for instance, for trait T5 (*h*^2^ = 0.62), GBLUP achieved an R^2^ of 0.4605, while for trait T3 (*h*^2^ = 0.40), it achieved an R^2^ of 0.2755.

The outperformance of our Transformer model under these conditions is therefore particularly telling. For trait T5, our model reached an R^2^ of 0.5527, and for T3, an R^2^ of 0.3291. Concurrently, we note that the performance gains, while consistent, are more modest for the low-heritability traits (MORT1 to MORT3). As detailed in Table 3, the standard deviations from our replicated runs confirm a stable, albeit smaller, advantage over baseline models. This is an expected outcome, as the high degree of phenotypic variation in these traits is influenced by non-genetic factors, fundamentally limiting the predictive ceiling for any model relying solely on genomic data. Nevertheless, our model’s ability to consistently outperform all baselines on these challenging traits underscores its enhanced capacity to capture the available genetic signals. This performance gap (R_Transformer_^2^ − R_GBLUP_^2^) suggests that the Transformer is successfully capturing a substantial portion of the non-additive genetic variance (dominance and epistasis) that contributes to the final phenotype but is invisible to linear models. This ability to learn and generalize these robust, heritable features—from local haplotypes to complex interactions—enables the model to more accurately predict the phenotypic outcome of a new generation based on its ancestors, thus excelling where kinship-based linear methods are inherently limited [13]. The crucial comparison with the “Transformer (no pre-train)” model further isolates and quantifies the substantial, additional benefit derived from the self-supervised pre-training phase [16,17].

### 4.2. Role of Self-Supervised Pre-Training

This research delivers both a methodological and an applied contribution to the field of computational breeding [3].

The methodological contribution lies in successfully introducing and validating the “pre-training and fine-tuning” paradigm, a cornerstone of modern natural language processing, for genomic selection in swine [15,16]. We demonstrate that leveraging genomic data in a self-supervised, unlabeled fashion is a powerful strategy for building superior predictive models [16,34]. This work provides a new, scalable, and transferable analytical framework that can be adapted for other species (e.g., cattle, poultry, crops) and other omics data types [18,23].

The applied contribution is of direct practical significance. The enhanced prediction accuracy translates directly to the potential for higher genetic gain [1,4]. By enabling more precise estimation of phenotypic values, our model allows breeders to make more accurate and earlier selection decisions [2]. This can accelerate the rate of genetic improvement for key economic traits, shortening breeding cycles and ultimately delivering significant economic benefits to the swine industry [1,35].

### 4.3. Interpretation and Biological Insights

While deep learning models are often termed “black boxes” [6,36], our framework offers new avenues for exploring the genetic architecture of complex traits. The self-attention mechanism, in particular, provides a direct window into how the model weighs the relationships between different genomic regions when predicting a phenotype.

To investigate the biological plausibility of the learned representations, we cross-validated the genomic regions assigned high attention weights by our model with the Pig QTLdb, a public database of quantitative trait loci [27]. As a specific example, for the Piglet Mortality (HZA-PMB) phenotype in the HZA-PMB dataset, we observed that the model identified a high-attention region on chromosome 13. This finding is highly consistent with published research, where previous studies have identified QTLs associated with the number of stillborn piglets on pig chromosomes 5 and 13 [28,29]. This alignment of model prediction with known biological markers strongly suggests that our model is not merely capturing statistical noise but is learning to identify genomic loci with known biological relevance to the phenotypic trait. Because our model operates on the complete genotype-to-phenotype map, these high-attention regions may represent not only loci with strong additive effects but also hubs involved in the epistatic networks that linear models cannot detect. This not only validates the model’s effectiveness but also demonstrates its potential as a powerful hypothesis-generation tool for discovering novel candidate genetic regions and interactions. While our primary analysis here focuses on attention weights, we acknowledge that other post-hoc interpretability techniques like SHAP or Integrated Gradients offer complementary approaches to quantify feature importance, which represent a valuable direction for future work [19,24].

### 4.4. Limitations and Future Directions

Despite the promising results, this study has several limitations that could be addressed, paving the way for future work.

First, while our model shows significant advantages, the absolute predictive accuracy for low-heritability traits like Piglet Mortality (HZA-PMB) remains modest. This is expected, as these traits are strongly influenced by complex non-additive genetic effects and, crucially, by large environmental factors not captured by SNP data alone [37]. The phenotypic variance is the sum of genetic and environmental variance. Any model using only genomic data is fundamentally limited by the proportion of variance that is not environmental. This highlights a general challenge in the field and an area for future improvement. A major avenue for improving predictive accuracy, especially for low-heritability traits, is the integration of multi-omics data and, critically, environmental data. Fusing genomics with transcriptomics, epigenomics, or even structured environmental variables (e.g., farm, season, diet) can provide a more holistic view of the factors driving the final phenotype [37].

Second, the interpretability of deep learning models, including ours, remains an active area of research. While the attention mechanism provides valuable insights into the model’s decision-making process by identifying high-attention genomic regions, more advanced post-hoc interpretability techniques, such as SHAP or Integrated Gradients, could offer a more granular and quantitative understanding of individual feature contributions [19]. A comparative analysis of different interpretability methods will be valuable to further demystify the model’s “black box” nature.

Third, we acknowledge that the computational cost of the self-supervised pre-training phase is substantial, requiring significant GPU resources. This might pose a barrier for some research groups. However, a key advantage of the “pre-train, fine-tune” paradigm is that the most intensive step needs to be performed only once.

Looking ahead, we propose several exciting research directions based on these limitations:

Integration of Multi-omics and Environmental Data: A major avenue for improving predictive accuracy, especially for low-heritability traits, is the integration of multi-omics data. Fusing genomics with transcriptomics or epigenomics can provide a more dynamic view of gene regulation [3].

Advanced Pre-training Objectives: Exploring alternative self-supervised tasks beyond masked prediction, such as contrastive learning, may enable the model to learn even more robust and informative genomic representations [16].

Advanced Interpretability and Biological Validation: Future work will focus on implementing and comparing advanced interpretability methods like SHAP. The insights gained will be systematically cross-validated with known biological information, such as QTL databases and gene ontologies. This will not only enhance the credibility of the model for applied users but also position it as a powerful hypothesis-generation tool for discovering novel candidate genes and biological pathways.

## 5. Conclusions

This study addresses the critical breeding challenge of enhancing genetic gain in swine [1] by tackling the fundamental problem of accurate phenotype prediction from genomic data. We fill a key methodological gap where existing methods like GBLUP fall short in capturing complex non-additive effects that contribute to phenotypic outcomes [4,7]. We successfully developed and validated a novel framework based on a self-supervised, pre-trained Transformer [13,16]. Experimental validation on real-world swine genomic datasets (PIC-GD and HZA-PMB) demonstrates that our model substantially outperforms baselines, including GBLUP and a Transformer trained from scratch [9,23]. This confirms our central scientific hypothesis that pre-training on the genomic data itself enables the model to learn intrinsic genomic structures, thereby boosting performance in the downstream task of phenotype prediction by capturing non-linear genetic signals [15,17,18].

The contributions of this research are twofold. As a methodological contribution, we provide a new, scalable deep learning framework for predicting complex phenotypes from high-dimensional genomic data [11,38]. As an applied contribution, our model directly serves to increase genetic gain in swine by delivering more accurate phenotype predictions, providing a powerful tool for more effective selection decisions in breeding programs [3,4].

Despite its strong performance, this work has limitations, including the need to validate its generalizability across more diverse populations and other species [39,40], while model interpretability remains a challenge [6,36]. Future work should focus on integrating multi-omics and environmental data to build more holistic predictive models [3], and advancing interpretability techniques to uncover the key genomic regions and interactions driving phenotypic variation, thereby bridging the gap between predictive power and biological insight [19,20].

## Figures and Tables

**Table 1:** Statistical summary of the datasets used (PIC-GD, HZA-PMB), including number of individuals, SNP markers, and key trait characteristics.

Statistic	PIC-GD	HZA-PMB
Number of Individuals	3534	8738
Genotyping Chip	Illumina PorcineSNP60	Illumina PorcineSNP60
SNPs (post-QC)	52,842	47,242
Traits Evaluated	5 Purebred Traits	Piglet Mortality (HZA-PMB) (3 Parities)
Trait Heritability ($h^{2}$)	0.07–0.62	0.06–0.11

**Table 2:** Key hyperparameter configurations for the Transformer model during pre-training and fine-tuning stages.

Hyperparameter	Pre-Training	Fine-Tuning
Transformer Layers (*L*)	12	12
Hidden Size ($d_{model}$)	768	768
Attention Heads (*H*)	12	12
FFN Intermediate Size	3072	3072
Dropout Rate	0.1	0.1
Optimizer	AdamW	AdamW
Learning Rate	1 × 10^−4^	5 × 10^−4^
Warmup Steps	100	10
Total Epochs	100	50

**Table 3:** Performance comparison ($R^{2}$) of different models across all traits for phenotype prediction, with the corresponding standard deviations in parentheses. All experiments were replicated five times to ensure the robustness of our findings. The best performance for each metric is highlighted in **bold**.

Model	T1	T2	T3	T4	T5	MORT1	MORT2	MORT3
	(h^2^ = 0.07)	(h^2^ = 0.25)	(h^2^ = 0.40)	(h^2^ = 0.55)	(h^2^ = 0.62)	(h^2^ = 0.063)	(h^2^ = 0.103)	(h^2^ = 0.114)
GBLUP	0.0512 (0.0025)	0.1859 (0.0026)	0.2755 (0.0052)	0.4058 (0.0072)	0.4605 (0.0097)	0.0418 (0.0016)	0.0815 (0.0026)	0.0921 (0.0030)
LASSO	0.0510 (0.0022)	0.1815 (0.0048)	0.2527 (0.0066)	0.4013 (0.0092)	0.4609 (0.0106)	0.0397 (0.0021)	0.0788 (0.0031)	0.0901 (0.0044)
LGBM	0.0517 (0.0019)	0.1863 (0.0045)	0.2780 (0.0050)	0.4062 (0.0084)	0.4624 (0.0092)	0.0426 (0.0025)	0.0823 (0.0038)	0.0917 (0.0032)
CNN	0.0523 (0.0021)	0.1871 (0.0034)	0.2801 (0.0064)	0.4085 (0.0078)	0.4638 (0.0107)	0.0431 (0.0027)	0.0827 (0.0030)	0.0926 (0.0035)
LSTM	0.0516 (0.0034)	0.1854 (0.0047)	0.2768 (0.0063)	0.4055 (0.0086)	0.4611 (0.0103)	0.0423 (0.0034)	0.0816 (0.0028)	0.0913 (0.0032)
Transformer (no pre-train)	0.0548 (0.0026)	0.2010 (0.0058)	0.3028 (0.0070)	0.4419 (0.0096)	0.5021 (0.0114)	0.0490 (0.0021)	0.0875 (0.0033)	0.0983 (0.0037)
**Transformer (with pre-train)**	**0.0574 (0.0022)**	**0.2161 (0.0053)**	**0.3291 (0.0072)**	**0.4855 (0.0093)**	**0.5524 (0.0111)**	**0.0535 (0.0020)**	**0.0904 (0.0031)**	**0.1020 (0.0039)**

## Data Availability

The raw genomic datasets (PIC-GD and HZA-PMB) analyzed during the current study are publicly available from their original publications, which are cited in the manuscript. The processed data used for model training are available from the corresponding author on reasonable request. All code for data preprocessing, model training, and analysis, along with the pre-trained model weights, will be made publicly available in a GitHub repository upon acceptance of the paper to ensure full reproducibility.
